# Single-Molecule Studies on a FRET Biosensor: Lessons from a Comparison of Fluorescent Protein Equipped versus Dye-Labeled Species

**DOI:** 10.3390/molecules23123105

**Published:** 2018-11-27

**Authors:** Henning Höfig, Michele Cerminara, Ilona Ritter, Antonie Schöne, Martina Pohl, Victoria Steffen, Julia Walter, Ignacio Vergara Dal Pont, Alexandros Katranidis, Jörg Fitter

**Affiliations:** 1 Forschungszentrum Jülich, ICS-5, 52425 Jülich, Germany; hoefig@physik.rwth-aachen.de (H.H.); michele.cerminara@gmail.com (M.C.); i.ritter@fz-juelich.de (I.R.); a.schoene@fz-juelich.de (A.S.); ignacioandres.vergara@med.uni-heidelberg.de (I.V.D.P.); a.katranidis@fz-juelich.de (A.K.); 2 RWTH Aachen University, I. Physikalisches Institut (IA), 52056 Aachen, Germany; walter@physik.rwth-aachen.de; 3 Forschungszentrum Jülich, IBG-1, 52425 Jülich, Germany; ma.pohl@fz-juelich.de (M.P.); victoria.steffen@uni-duesseldorf.de (V.S.)

**Keywords:** Förster resonance energy transfer (FRET), single molecule studies, biosensor, fluorescent protein (FP), conformational change, hinge motion, ligand binding, glucose sensor

## Abstract

Bacterial periplasmic binding proteins (PBPs) undergo a pronounced ligand-induced conformational change which can be employed to monitor ligand concentrations. The most common strategy to take advantage of this conformational change for a biosensor design is to use a Förster resonance energy transfer (FRET) signal. This can be achieved by attaching either two fluorescent proteins (FPs) or two organic fluorescent dyes of different colors to the PBPs in order to obtain an optical readout signal which is closely related to the ligand concentration. In this study we compare a FP-equipped and a dye-labeled version of the glucose/galactose binding protein MglB at the single-molecule level. The comparison demonstrates that changes in the FRET signal upon glucose binding are more pronounced for the FP-equipped sensor construct as compared to the dye-labeled analog. Moreover, the FP-equipped sensor showed a strong increase of the FRET signal under crowding conditions whereas the dye-labeled sensor was not influenced by crowding. The choice of a labeling scheme should therefore be made depending on the application of a FRET-based sensor.

## 1. Introduction

The diversity of biological functions, like ligand binding, conformational changes, or structural adaptability of proteins is used already since many years to engineer biosensors [1]. In particular bacterial periplasmic binding proteins (PBPs) have been exploited to develop metabolite sensors [2]. Typically, PBPs consist of two domains connected by a hinge region which includes a ligand binding site located at the interface of the two flanking domains. Depending on the ligand binding status the whole structure can adopt two different conformations: a ligand-free open form and a ligand-bound closed form, which interconvert through a bending and a swiveling twist motion about the hinge [2,3,4,5]. First approaches to make use of PBPs as biosensors were based on site-specific coupling of environmentally sensitive extrinsic fluorophores to the domain interface of the PBP. Ligand binding was reported as changes in fluorescence intensity, for example by local quenching effects. Often these quenching effects are related to large changes in solvent accessibility of the fluorophores between the ligand-free open state and the ligand-bound closed state [5].

Later for in vivo applications the PBP-based sensors were equipped with two fluorescent proteins (FPs) that utilize Förster resonance energy transfer (FRET) to measure the ligand-induced conformational changes. For this purpose, variants of an appropriate FRET donor and acceptor pair, e.g., a cyan FP (donor) and a yellow FP (acceptor), were fused to the amino- and carboxyl-termini of the central ligand binding domain, respectively. Conformational changes of this sensing domain induce a change in the distance and/or the relative orientation of the FPs, which ultimately causes alterations of the FRET efficiency in a ligand concentration-dependent manner [6,7,8]. These genetically encoded FRET biosensors can monitor steady-state levels of ions or metabolites in cells, for example to compare differences between mutants and wild types [9,10]. Therefore, genetically encoded fluorescence sensors have become essential tools in modern biological research and many recent advances have expanded the scope of applications [1].

The design and optimization of genetically encoded FRET sensors is often a challenging task, since the change in the optical readout signal upon alteration of the ligand concentration needs to be sufficiently large [6,7,10,11,12]. The major challenge for FRET-based biosensors, which make use of the Venus flytrap principle [3], is given by the fact that a conformational change in the sensing domain must be translated into a significant variation of the relative distance and/or orientation of the attached FPs. At first glance the use of rather bulky FPs attached to the terminal ends of the sensing domain seems not to be a very promising approach to sense the conformational changes induced by the bound ligand. In practice though, the empirical screening of libraries of linker variants inserted between the sensing domain and the attached FPs have produced sensor constructs with dramatically improved signal changes [6,12]. However, this is often still a trial and error approach in which many constructs of various linker lengths, sensing domains and FPs need to be tested in order to obtain a sufficiently large response signal [11]. 

An alternative approach makes use of sensing proteins (e.g., PBPs) to which a pair of small organic fluorescent dyes is attached at suitable positions in the protein sequence. These constructs are again employed as FRET sensors, in which the dye attachment is chemically performed at cysteine residues that were genetically introduced at the desired positions (see for example [13,14,15]). However, the fluorescent dye-based approaches are generally hard to apply for in vivo measurements. 

In this work we report on a comparison between a dye-labeled and a FP-based FRET biosensor and discuss advantages and disadvantages of both labeling schemes, as well as their potential for different applications. As a case study we compared a FP-equipped and dye-labeled FRET-based sensors for glucose [6,16] that use the same glucose/galactose binding protein (MglB) from *Escherichia coli*. We employ single-molecule FRET (smFRET) analyses to compare the response of the respective sensors to glucose and crowding conditions.

## 2. Results

### 2.1. Design of the Glucose Binding Protein for Site-Specific Dye Attachement

In order to find the best design of a dye-based FRET sensor that monitors the glucose-induced conformational changes within the glucose binding protein (MglB), two 3D-structures from the Protein Data Bank (PDB) were considered; the ligand-free (2FW0) and the ligand-bound form (2FVY). For the intended smFRET studies we employed Alexa Fluor 488 (AF488) as a donor dye and Alexa Fluor 647 (AF647) as an acceptor dye with a corresponding Förster radius of R_0_~54.5 Å (see Section Materials and Methods for further details). Since an inter-dye distance R_DA_ close to the Förster radius is most sensitive to conformational changes induced by glucose binding, two surface accessible positions for dye attachment had to be identified which: (i) exhibit an averaged inter-dye distance close to R_0_ concomitant with (ii) a large difference in R_DA_ between the ligand-free and the ligand-bound structures. In addition to these conditions required for a large FRET signal change, the biological function of the protein should not be affected by the amino acid exchanges needed to attach the fluorescent dyes, which means that the substituted amino acids should not be critical for the structure and for the ligand binding of the protein.

**Figure 1:**
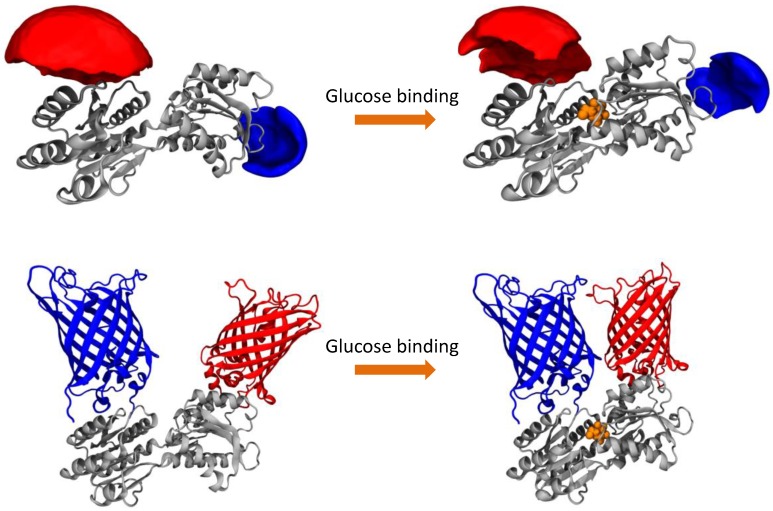
(upper panels) Illustration of chosen dye labeling positions to design a FRET-based glucose sensor: 3D structures of ligand-free (left structure; 2FW0) and ligand-bound MglB (right structure; 2FVY) shown together with calculated AVs for donor (blue volumes, attached at position 137) and acceptor (red volumes, attached at position 42) dyes. Note the conformational change of the MglB structure induced by glucose (orange spheres) binding. (lower panels) For comparison, an equivalent illustration of both MglB structures is shown with donor FPs (blue, inserted at position 12) and acceptor FPs (red, inserted at the C-terminus). The relative orientations of the FPs are arbitrary and not based on experimental measures.

As a good choice in this respect cysteine residues were introduced at positions 42 and 137 (for details see Materials and Methods), as shown in Figure 1. In order to guarantee sufficient rotational flexibility (relevant to fulfill the κ^2^~2/3 criterion), the attached dyes were bound to the cysteines via C6 amino linkers (NH_2_-C_6_H_12_-NH_2_) which enable additionally dye linker dynamics. This translational mobility is represented by the accessible volumes (AV) of both dyes which can be estimated by an appropriate algorithm [17]. 

As demonstrated in various studies the combination of all possible dye positions calculated by these AVs give the most reliable average inter-dye distances [18,19], which we call $R_{DA}^{AV}$.

**Figure 2:**
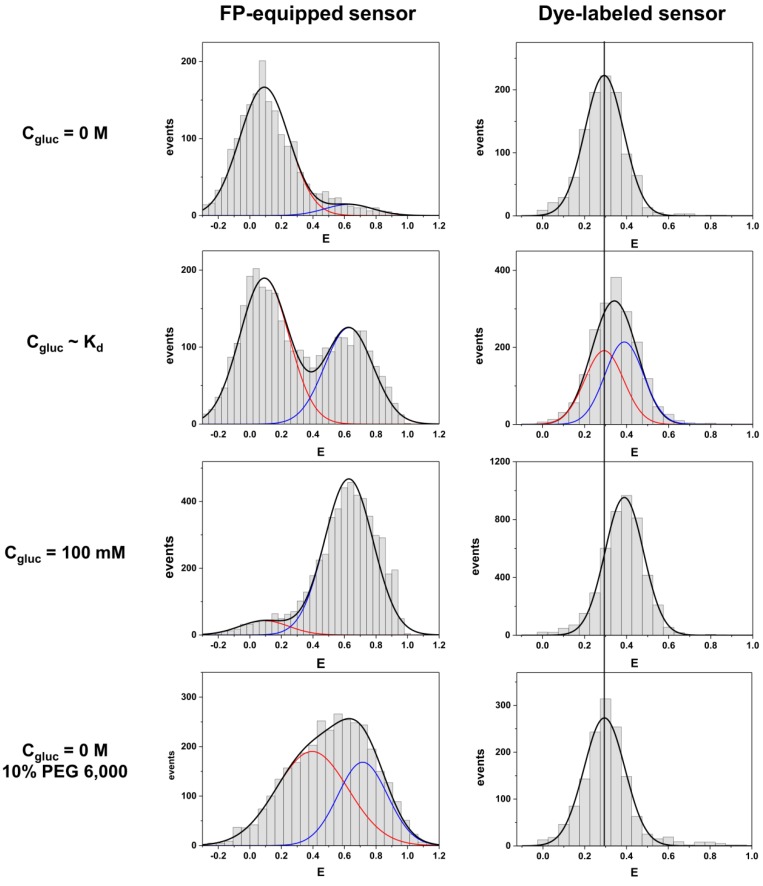
Comparison of smFRET histograms obtained from FP-equipped (left column) and dye-labeled (right column) species of MglB for four different conditions (from top to bottom): ligand-free state, ligand-bound state at the concentration near the K_d_ (for values see Figure 3), fully ligand-bound state and ligand-free state in presence of 10% (*w*/*w*) PEG 6,000 as a crowding agent. Data shown for the FP-equipped sample were taken from [22]. For the sample labeled with fluorescent dyes a vertical line corresponding to the position of the ligand-free state, is drawn for reference.

Table 1 also shows the often used C_α_—positions of the introduced cysteines giving distance values $R_{DA}^{C_{\alpha }}$ which deviate significantly from the $R_{DA}^{AV}$-values. The inter-dye distance values based on the AV-calculations give calculated energy transfer efficiency values (*E* ^calc^) which are in reasonable agreement with the E-values obtained from the experimental data (see Figure 2). As shown in Table 1 the chosen dye-attachment positions give shorter R_DA_-values for the ligand-bound state compared to the ligand-free state. For measuring structural differences as a function of glucose concentration, it is most important that the expected differences in the E-values (i.e., ΔE~0.13) are large enough to be detectable from smFRET histograms. 

### 2.2. Comparison of FP-Equipped and Dye-Labeled Sensors on the Basis of smFRET Histogram

smFRET studies conducted on proteins labeled with bright fluorescent dyes is an established approach which was used already in many applications (see for example [20,21]). In contrast, similar smFRET studies with FP-equipped proteins are more challenging, mainly due to the significantly less photo-stable FPs as compared to organic dyes. Only recently we demonstrated the feasibility of such a smFRET study with reasonable counting statistics. We studied several sensor constructs that contain MglB as the sensing domain and were equipped with mTurquoise2 as a donor and Venus as an acceptor FP. The constructs exhibit changes in energy transfer efficiencies (i.e., ΔE) that differ drastically, depending on the type of linker variants inserted between the FPs and the sensing domain MglB [22]. For several sensor constructs smFRET histograms were obtained without glucose, at glucose concentrations near the sensor’s K_d_, and at glucose concentrations where the sensor was fully saturated. 

Here we present a comparison of smFRET data obtained from a dye-labeled MglB species with that from an FP-equipped construct (see Figure 2). We chose a FP-equipped sensor construct with a very good sensing performance (construct no. 2 as described in [22]) and observed pronounced differences compared to the dye-equipped sensor. While the FP-equipped species exhibits two clearly separated populations for the ligand-free and the ligand-bound state, which are separated by a value of ΔE~0.53, the corresponding value for the dye-equipped sample exhibits only a ΔE~0.1 (see Figure 2). With respect to the distribution width of each population, the dye-equipped sample does not show two clearly separated peaks corresponding to the ligand-free and ligand-bound states, but rather an apparent single peak of the merged populations, whose position shifts with the ligand concentration (for further details see Appendix A). Due to rather long measuring times for FP-equipped samples needed to obtain reasonable counting statistics, feasible smFRET histograms are only available for three different glucose concentrations (namely without ligand, around K_d_ and under ligand-saturated conditions, as shown in Figure 2). For the dye-equipped sensor we measured at sixteen different conditions, covering the glucose concentration regime between 0 and 125 mM.

**Figure 3:**
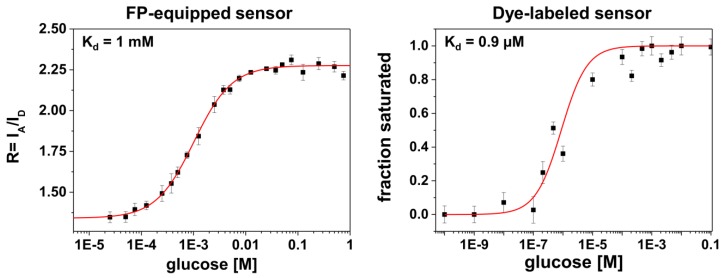
FRET parameter as a function of glucose concentration for the two kinds of MgIB-based sensors. For the FP-equipped constructs ensemble data were used (taken from [22]) to calculate a FRET ratio R = I_A_/I_D_ (where I_A_ and I_D_ are the peak intensities of the acceptor and the donor emission, respectively) (left panel), while for the dye-labeled construct the smFRET statistical weights of the liganded population are reported (right panel).

Two histograms of the dye-labeled sensor were fitted with a single population: in the absence of glucose (ligand-free state with peak position at E~0.29) and at 100 mM glucose (fully ligand-bound state with peak position at E~0.39). All other histograms with concentrations between these values were fitted with two Gaussians, with fixed center positions and widths corresponding to the two limiting cases mentioned before. From these fits statistical weights for each component were obtained from the area under the Gaussian curve. A plot of statistical weights related to the ligand-bound population (centered at E~0.39) against the glucose concentration is shown in Figure 3 (see right panel). For the FP-equipped sensors we consider similar data from ensemble FRET (data from [22]) due to the experimental limitations explained above. In both cases a sigmoidal binding curve was fitted to the experimental data points. Although we used the same sensing domain (MglB) for both constructs the binding curves exhibit very different half-maximal rise values, which represent the dissociation constant K_d_ of the glucose binding to MglB. While the dye-labeled construct shows a K_d_ value of about ~0.9 μM, the FP-equipped sensor exhibits a much larger value in the order of 1 mM.

Another clear difference between the sensors with different fluorophores is given by their response to crowding conditions. We incubated both types of sensors in the ligand-free state (i.e., 0 M glucose) in a buffer solution enriched by a high concentration of polyethylene glycol with a molecular mass of 6000 Dalton (PEG 6000). PEG polymers are often used as synthetic crowding agents to mimic cellular crowding [23]. As we pointed out in an earlier publication [22], FP-equipped sensor constructs experience a pronounced structural compaction upon crowding. This causes a shift of all populations towards larger energy transfer efficiencies in the smFRET histogram (Figure 2). In addition, a partial depopulation of the low FRET population and a concurrent additional population of the high FRET state takes place also in the absence of glucose. In contrast, the dye-equipped species is not sensing any compaction by the crowding agent. The obtained smFRET histogram remained almost unchanged as compared to the pure buffer condition. 

## 3. Discussion

To the best of our knowledge we show here for the first time a direct comparative smFRET study of two optimized biosensors that are based on the same sensing protein equipped either with a pair of FPs or with a pair of fluorescent dyes. In particular, we compared an extensively optimized FP-equipped glucose sensor construct based on the glucose/galactose binding protein MglB with a dye-labeled MglB where the attachment positions were optimized to achieve a large FRET signal change. In contrast to ensemble data, single-molecule data provide detailed information on coexisting sensor subpopulations, which in addition permits the discrimination of heterogeneities arising from experimental artifacts or impaired sample preparation, including effects of incomplete FP chromophore maturation [22,24]. In this respect it is worth to mention that our smFRET approach allows the elimination of donor-only molecules which would otherwise bias the resulting FRET efficiencies drastically [22]. Therefore, the smFRET approach is ideally suited to perform a rigorous comparison of both labeling schemes in order to judge the respective performances.

We compare both labeling schemes by means of FRET efficiency histograms because the energy transfer efficiency is the underlying physical parameter in contrast to derived parameters such as emission intensity ratios. The most obvious difference between the two labeling schemes is given by the fact that the dye-equipped sensor exhibits a much smaller energy transfer change between the ligand-free and the ligand-bound state as compared to the FP-equipped sensor. This is mainly caused by the fact that the dye-equipped sensor can only make use of changes in the inter-dye distance R_DA_ (see Table 1). The extrinsic fluorophores are usually attached to the protein via a long and flexible linker, to avoid the interaction of the dyes with the surface of the protein, which could give rise to a quenching of the fluorescence, and to guarantee a fast and relatively free rotation of the dyes (see AVs in Figure 1). As a consequence, the orientation factor that modulates the FRET efficiency can be approximated to a constant value of 2/3 and the only factor influencing the FRET efficiency is the inter-dye distance. The inter-dye distance change ΔR_DA_ is physically restricted by the absolute conformational change of the sensing proteins. For sensing proteins with sizes in the order of 30–35 kDa (like PBPs) or smaller, the conformational change is rather moderate, which remains an inherent problem. In addition, only attachment positions for which R_DA_ is close to the Förster radius R_0_ can be used in order to be sensitive to distance changes with FRET. Although we could identify attachment points on the surface of the MglB protein which would result in larger ΔR_DA_ values, they are not favorable because their R_DA_ values are too small with respect to the actual R_0_. However, even if we would consider the largest possible ΔR_DA_ value with the best matching R_0_, the difference in transfer efficiency would still be significantly smaller than the value of the FP-equipped sensor.

In contrast, FP-equipped sensors are designed in a way that other factors rather than only the distance change ΔR_DA_ between both FPs contribute to the FRET signal change upon ligand binding. These approaches try to make use of a restriction of the spatial orientations occupied by the FPs. Thereby the orientation parameter κ^2^ can be the dominant contribution to the FRET efficiency, which helps to increase the FRET signal change [6,7,12]. In contrast to AV calculation for dyes, the problem for FP-based sensors is still to predict possible fluorophore orientations for a specific design, although there are some approaches reported in the literature [25]. As a consequence, often many constructs with a variety of insertion positions of FPs and linker properties have to be tested. In most of these attempts at least a few of the tested constructs show a reasonable performance [12,22].

Another feature in the comparison between the different labeling schemes is given by rather different apparent binding affinities (in terms of K_d_-values, see Figure 3). It is known from previous studies that for FP-equipped as well as for dye-equipped sensors the binding affinity can be drastically reduced compared to the K_d_ of the isolated glucose/galactose binding protein (MglB) [5,26,27]. On the one hand, we observe a K_d_ value of ~1 mM for the FP-equipped construct which is rather close to that of the starting construct (FLII^12^Pglu600µ) [6]. On the other hand, our dye-labeled sensor construct exhibits a K_d_ value of ~0.9 μM which is close to that of the wild-type MglB [26]. Since for both sensor constructs almost identical MglB binding proteins were employed, the drastically reduced binding affinity of the FP-equipped sensor can be attributed to an impact predominantly originating from the FP attachment/insertion to/into the binding protein itself. In this respect the attachment of rather bulky FPs to the binding protein may alter the ligand accessibility of the binding site or modifies the well-balanced atomic structure of the binding site directly by FP induced strain.

The last pronounced difference between both sensors is related to their response to macromolecular crowding. Only the FP-equipped sensor exhibited a pronounced compaction in the presence of PEG 6000, while the dye-labeled species did not. This indicates that the volume expansion of the sensing protein alone is not much affected by this crowding agent. Interestingly, our observation somehow disagrees with recent findings which claim some PEG-induced compaction of the glucose binding protein [28]. Most probably this disagreement is caused by the rather different experimental parameters which were measured in both studies. While smFRET measures the real physical dimension of the sensing protein, intrinsic protein fluorescence (emission wavelength shifts or altered emission intensities of intrinsic tryptophan residues) gives only nonspecific information about a change in the local environment of the tryptophan residues. Although for practical reasons genetically encoded FRET-based sensors are used to measure metabolite concentrations in cells, they would nevertheless require a specific calibration to consider crowding effects, which is a challenging task. In addition to ligand binding, also cellular crowding induces a structural compaction and thereby a FRET signal change which would bias the read out. An approach to analyze both effects separately is possible by comparing ordinary sensors to sensor variants with disabled ligand binding sites [29]. 

Our comparison of two FRET-based glucose sensors reveals that the signal change of the FP-equipped sensor outperforms that of the dye-labeled analog. The attachment positions of the fluorophores on the surface of the glucose binding protein MglB vary for both sensors because both are optimized to reach a large signal change upon glucose binding. However, the choice of a sensor type may consider other aspects depending on the application of the sensor. The dye-labeled sensor may be beneficial if a high affinity is desired. Yet, the major advantage of the dye-labeled sensor is its insensitivity towards crowding. Thus, the application of dye-equipped sensors in living cells is a possible alternative using microinjection [30], although this procedure is rather time-consuming. It can be likewise beneficial to use FP-equipped sensors for in vitro applications because of their large signal changes [12,16].

Finally, the transfer of our findings to other biosensors based on the family of periplasmic binding proteins (PBP) depends on two factors: (i) how extensively has the respective FP-equipped sensor already been optimized and (ii) are dye labeling positions of the respective PBP available that result in large distance changes in the range of the Förster radius. The latter can in principle be predicted ab initio, if high resolution crystal structures are known for the liganded and non-liganded state, as we have demonstrated here with the case study of MglB. 

## 4. Materials and Methods 

### 4.1. DNA Constructs 

The gene encoding the glucose sensor FLII^12^Pglu600µ, which consists of the glucose/galactose-binding protein MglB from *E. coli* and the fluorescent proteins (FPs) ECFP and Citrine (EYFP with Q69M), was cloned between the NdeI and HindIII sites of the pRSET vector [6]. The FPs were altered to the improved variants mTurquoise2 and Venus, respectively. mTurquoise2 was inserted after the 11 N-terminal amino acids of MglB to make it more rigid and Venus was fused to the C-terminal end as previously described [22]. For sequence information see glucose sensor no. 2 in Supporting Information of ref. [22]. For the dye-labeled sensor the MglB protein was amplified from the above mentioned construct and point mutations Q42C and K137C were introduced at the same time. The amino acid sequence of this construct is given in Appendix B. The resulting PCR product was cloned between the NdeI and XhoI sites of the pRSET vector.

### 4.2. Production and Purification of MglB 

The variant MglB Q42C K137C with C-terminal hexahistidine tag was produced in *E. coli* BL21 RIL cells for 3 h at 37 °C after induction with 1 mM isopropyl-β-d-thiogalactopyranoside (IPTG) at an optical density (O.D.) of 0.6. The cells were harvested and resuspended in buffer A containing one tablet of EDTA-free protease inhibitor (cOmplete Ultra, Merck, Darmstadt, Germany). Cells were lysed using a cell disruptor (Constant Systems Ltd., Daventry, UK). The cell lysate was filtered (pore size 0.2 µm) and applied to a Ni-NTA affinity column (Qiagen, Venlo, the Netherlands) equilibrated with buffer A (20 mM MOPS, 300 mM NaCl, pH 7.3). After washing with buffer A containing 10 mM and 20 mM imidazole, MglB was eluted with 250 mM imidazole. The protein was desalted in a Superdex 200 10/300 GL size exclusion column (GE Healthcare Life Sciences, Chicago, IL, USA) with 20 mM MOPS, pH 7.3, flash frozen and stored at −80 °C for further use. 

### 4.3. Dye Labeling of the MglB Protein 

The MglB protein was labeled on the two cysteine residues at positions 42 and 137 using approximately 5-fold excess of the acceptor dye Alexa Fluor 647 (AF647) and the donor dye Alexa Fluor 488 (AF488) in a 1.5:1 ratio. The excess of unbound fluorophores was removed by a Superdex 200 10/300 GL size-exclusion column. The double-labeled protein was purified via a MonoQ 5/50 GL ion-exchange column (GE Healthcare Life Sciences, Chicago, IL, USA) in an automated FPLC system (ÄKTAexplorer, GE Healthcare Life Sciences, Chicago, IL, USA) using a continuous NaCl gradient (0–500 mM NaCl) in 20 mM MOPS, pH 7.3. The different species were separated according to their charge due to the negative net charge of the bound fluorophores at pH 7.3. The obtained label stoichiometry is given by 43% donor only, 21% acceptor only, and 36% donor and acceptor labeled species. Since all smFRET measurements were performed using Pulsed Interleaved Excitation (PIE), we did not improve the label efficiency in this study.

### 4.4. smFRET Measurements with AF488 and AF647 Labeled MglB Sensors 

A detailed description of smFRET measurements was published earlier [15,31]. We performed our studies with diffusing double labeled sensor constructs using a confocal inverted microscope MicroTime200 (PicoQuant, Berlin, Germany). Shortly, the fluorophores were excited using lasers with 485 nm and 640 nm (LDH-D-C 640B and LDH-D-C 485B from PicoQuant, Berlin, Germany), respectively. The excitation light was focused on the sample using a high numerical aperture water immersion objective (UPLSAPO 60x; Olympus, Hamburg, Germany); the fluorescence emitted was collected through the same objective and spatially filtered by a 75 μm pinhole in confocal configuration. The emission signal was separated by a dichroic mirror (T600lpxr, Chroma Technology, Olching, Germany) and filtered by a 535 nm band pass filter (535/55, Semrock, Rochester, NY, USA) for the donor channel and a 635 nm long-pass filter (635 LP, Semrock, Rochester, NY, USA) for the acceptor channel. Finally, photons were detected by single-photon avalanche diodes (τ-SPAD, PicoQuant, Berlin, Germany for the donor channel and SPCM-AQR-14, Perkin-Elmer Inc., Waltham, MA, USA for the acceptor channel). The arrival time of each photon was recorded with a time-correlated single-photon counting module (HydraHarp400, PicoQuant, Berlin, Germany). For smFRET, a PIE scheme was applied, in which excitation of the donor and acceptor is alternated in order to verify the presence of the acceptor by direct excitation and minimize the artifacts due to the presence of donor-only molecules. All measurements were made in presence of 0.001% Tween20 and a Trolox-cysteamine cocktail was used for photo-protection purposes. 

### 4.5. Accessible Volume (AV)-Calculations and Data Analysis 

The AVs for fluorescent dyes attached to MglB were determined using an algorithm that was previously described by Höfig et al. [17]. In brief, this algorithm generates for each dye bound to the protein an AV cloud, where each point is sterically accessible for the fluorophore. This algorithm takes the length of the linker and the shape of the dyes into account as well as the fact that the dye is free to move around the anchor position. By averaging all the possible positions for both dyes, a mean donor-acceptor distance is calculated.

Analysis of smFRET data was performed using self-written Matlab routines (Mathworks, Natick, MA, USA), whose details are described in Gabba et al. [15]. Briefly, the bursts (i.e., the photons emitted by the dyes when a labeled protein crosses the confocal volume) were identified from the raw data according to the inter-photon lag-time between two adjacent photons, which has to be below a certain threshold. This first selection was done on the photons emitted upon direct excitation of the acceptor, therefore ensuring the presence of this dye and in turn avoiding the selection of protein labeled only with the donor. To the bursts selected upon direct excitation of the acceptor, a further criterion was applied. Here, only the bursts having a total number of photons (donor + acceptor) emitted upon excitation of the donor (FRET photons) above a threshold value were considered for the analysis. According to established standard procedures, for each burst the number of photons emitted from the donor and from the acceptor were counted and the corresponding fluorescence intensities were calculated by correcting for the background photon counts, the crosstalk of the donor fluorescence into the acceptor channel, the detection efficiencies for the two channels, and for the different QY of the dyes [24]. For each such selected burst, donor (D) and acceptor (A) photons after direct donor excitation were accumulated and corrected:

(1) $$F_{D}=I_{D}-BG_{D}$$

(2) $$F_{A}=I_{A}-BG_{A}\;-\;Lk\;-\;Dir$$

Here, *F_D_* and *F_A_* are the corrected intensities of donor and acceptor, *I_D_* and *I_A_* are the raw data of donor and acceptor counts, and *BG_D_* and *BG_A_* are the background counts of donor and acceptor obtained as the product of dwell time and average background count rate, respectively. *Lk* represents the leakage of donor photons into the acceptor channel and *Dir* gives the acceptor photons originating from direct excitation. For all bursts that fulfilled a threshold of *F_D_*+ *F_A_* > 25, the energy transfer efficiency:

(3) $$E=\frac{F_{A}}{F_{A}+\gamma \cdot F_{D}}$$

was calculated, where γ is a correction factor defined as:

(4) $$\gamma =\frac{\Phi_{A}}{\Phi_{D}}\cdot \frac{g_{A}}{g_{D}}$$

Here, Φ_A_ and Φ_D_ are the fluorescence quantum yields while *g_A_* and *g_D_* are the transmission efficiencies of the acceptor and donor, respectively. For the calculation of expected energy transfer efficiency values from inter-dye distances R_DA_ known from the 3D protein structures, the relation:

(5) $$E\frac{1}{1+{\left(\frac{R_{DA}}{R_{0}}\right)}^{6}}$$

is used, with a Förster radius of R_0_ = 54.5 Å for the conditions used here.

### 4.6. Production, Measurements and Data Analysis of FP-Equipped MglB Constructs

All details about the design, production, and purification of FP-equipped sensor constructs are described in a recently published work [22]. In this publication also all ensemble and smFRET measurements of the construct no. 2, the construct which we discuss in this work, are described in detail. With the help of two-color coincidence detection (TCCD) we also determined the label stoichiometry of the sensor construct. The sensor no. 2, for which data are presented here, is characterized by 31% donor only, 9% acceptor only, and 60% donor and acceptor labeled constructs (see ref. [22]). These values are essentially determined by the chromophore maturation efficiency of the individual FPs within the given sensor construct.

## Figures and Tables

**Table 1:** smFRET parameters calculated on the basis of the MglB crystal structures from the ligand-free and ligand-bound state (see Figure 1).

Sample	$R_{DA}^{C_{\alpha }}$	$R_{DA}^{AV}$	*E* ^calc 1^
PDB entry 2FW0 (ligand-free)	50 Å	65 Å	0.27
PDB entry 2FVY (ligand-bound)	45 Å	58 Å	0.40

^1^*E*^calc^—values were calculated on the basis of $R_{DA}^{AV}$—values, see Equation (5) in Section Materials and Methods.

**Figure A1:**
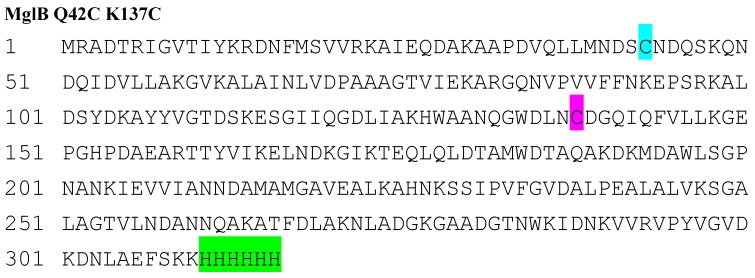
Amino acid sequence of the MglB protein is given as used in this study. The two inserted cysteine residues at positions 42 and 137 are highlighted cyan and purple, respectively. The C-terminal His-tag is highlighted green.

## Appendix A

**Table A1:** Overview on the parameters obtained from histogram fittings of the dye-equipped MglB glucose sensor under different conditions as shown in Figure 2. E_1_ and E_2_ represent the means of the energy transfer efficiency values of the two resolved populations, σ_1_ and σ_2_ are the related widths, and p_1_ and p_2_ are the statistical weights of the first and second Gaussian, respectively. The “# Events” give the number of bursts which were considered to build the respective histogram.

Sensor Conditions	E_1_	E_2_	σ_1_	σ_2_	p_1_	p_2_	# Events
0 M glucose	0.29	-	0.092	-	-	-	1060
1 μM glucose	0.29	0.39	0.092	0.092	0.47	0.53	1897
100 mM glucose	0.39	-	0.092	-	-	-	4555
10% PEG 6000	0.29	-	0.098	-	-	-	1422
